# Cultural stereotypes as gatekeepers: increasing girls’ interest in computer science and engineering by diversifying stereotypes

**DOI:** 10.3389/fpsyg.2015.00049

**Published:** 2015-02-11

**Authors:** Sapna Cheryan, Allison Master, Andrew N. Meltzoff

**Affiliations:** ^1^Department of Psychology, University of WashingtonSeattle, WA, USA; ^2^Institute for Learning & Brain Sciences, University of WashingtonSeattle, WA, USA

**Keywords:** science, underrepresentation, belonging, gender, stereotypes

## Abstract

Despite having made significant inroads into many traditionally male-dominated fields (e.g., biology, chemistry), women continue to be underrepresented in computer science and engineering. We propose that students’ stereotypes about the culture of these fields—including the kind of people, the work involved, and the values of the field—steer girls away from choosing to enter them. Computer science and engineering are stereotyped in modern American culture as male-oriented fields that involve social isolation, an intense focus on machinery, and inborn brilliance. These stereotypes are compatible with qualities that are typically more valued in men than women in American culture. As a result, when computer science and engineering stereotypes are salient, girls report less interest in these fields than their male peers. However, altering these stereotypes—by broadening the representation of the people who do this work, the work itself, and the environments in which it occurs—significantly increases girls’ sense of belonging and interest in the field. Academic stereotypes thus serve as gatekeepers, driving girls away from certain fields and constraining their learning opportunities and career aspirations.

In 2010, Mattel let girls vote online for which career they wanted Barbie to have next. They gave girls a choice of one of five careers: news anchor, architect, surgeon, environmentalist, and computer engineer. Computer Engineer Barbie ended up winning by a landslide after female engineers and others in technology launched online campaigns in technology communities to get out the vote. Their hope was that future generations of girls would play with Computer Engineer Barbie and be inspired to pursue careers in computer science and engineering (Martincic and Bhatnagar, 2012). After the voting closed, Mattel announced the simultaneous release of two of the Barbies: Computer Engineer and News Anchor. Although Computer Engineer Barbie had won the “popular vote,” Mattel’s empirical research showed that the “girls’ vote” went to News Anchor Barbie (Zimmerman, 2010). This anecdote is symbolic of a broader trend in our society: despite efforts by people in education, technology, government, and non-profits to get girls interested in a future in computer science and engineering, girls are choosing other fields.

Women currently make up 48% of medical school graduates and 47% of law school graduates (Jolliff et al., 2012; American Bar Association, 2014). Even within STEM (science, technology, engineering, and math), women obtain the majority of the U.S. undergraduate degrees (59%) in biology and nearly half in chemistry and math (National Science Foundation, 2013). However, in computer science and engineering, women earn less than 20% of undergraduate degrees (National Science Foundation, 2013). Gender disparities in computer science and engineering are problematic for at least three reasons. First, jobs in these fields are often high-status, lucrative, and flexible (Kalwarski et al., 2007), and thus women are missing out on jobs that are potentially beneficial for them. Second, computer scientists and engineers design tools that shape modern society, and diversifying the field can help to ensure that these fields are creating designs appropriate for a broad population (Margolis and Fisher, 2002). Third, the U.S. is currently not training enough computer scientists and engineers to keep up with demand (Soper, 2014). Attracting more women and people of color would be an effective way of reducing this gap.

Women have entered many other previously male-dominated fields, including other STEM fields, but not computer science and engineering. Why the differential? According to Gelernter (1999), professor of computer science at Yale, the explanation for women’s underrepresentation is obvious, “Women…must be choosing not to enter, presumably because they don’t want to; presumably because they (by and large) don’t like these fields.” His statement assumes that women’s choices are freely made and not constrained. If women are freely choosing not to pursue computer science, perhaps nothing can or should be done about it—after all, it is their choice. However, it is clear from a large body of scientific research that there are significant social barriers to women’s entry into computer science and engineering that preclude women from being able to make a truly “free” choice (Ceci et al., 2009). Here we analyze those barriers and what can be done about them.

In what ways are girls’ educational choices constrained? First, girls may be steered away from computer science and engineering by parents, teachers, and others who think that these careers are better suited for boys (Eccles et al., 1990; Sadker and Sadker, 1994). Second, the mere fact of having underrepresentation can perpetuate future underrepresentation (Murphy et al., 2007). If girls do not see computer scientists and engineers as people with whom they feel similar, they may be more reluctant to enter these fields (Dasgupta, 2011; Meltzoff, 2013). Third, girls systematically underestimate how well they will do in these fields, and this predicts their lower interest in entering them (Correll, 2001; Ehrlinger and Dunning, 2003). Fourth, girls may anticipate encountering greater work-family conflicts in these fields (Ceci et al., 2009). Fifth, there is discrimination in these fields that prevents qualified women from receiving the same opportunities as their male counterparts (Moss-Racusin et al., 2012). Sixth, women who enter traditionally masculine domains can be socially and professionally penalized for exhibiting competence and leadership qualities (Rudman, 1998). These are all barriers that contribute to why some women choose not to enter and persist in fields like computer science and engineering. Note, however, that these barriers previously existed (and continue to exist) in other male-dominated fields that women have entered. A key question remains: *what has allowed other fields to welcome more women while computer science and engineering continue to lag behind?*

In this paper, we present evidence for a novel and powerful social factor perpetuating the underrepresentation of women and girls: stereotypes about the culture of these fields. We begin by differentiating *stereotypes about the culture* from the large body of useful work on *stereotype threat*. Then, we describe the content of students’ stereotypes about the culture of computer science and engineering and document their pervasiveness in the minds of American students. Third, we describe three ways that these stereotypes about the culture are transmitted: through environments, the media, and the people in the fields, and why these stereotypes are a more powerful deterrent for girls than boys. Fourth, we present empirical evidence that these stereotypes cause gender disparities in interest in entering computer science and engineering not only in college but earlier in the pipeline, including among high-school students. Finally, we show that these stereotypes, while powerful, are nonetheless highly malleable and that changing them encourages girls and women to enter these fields (without dissuading boys and men). Note that research on different populations, at different ages, and asking different questions (e.g., why are women underrepresented in the STEM workforce?) may discover different factors responsible (e.g., Eagly and Carli, 2007; Hewlett et al., 2008; Ceci et al., 2009, 2014). Our argument is that stereotypes of the field act as educational gatekeepers, constraining who enters these fields, and that interventions to broaden the cultural representation of these fields can help to draw more diversity into them.

## DUAL STEREOTYPES AND GENDER DISPARITIES

**FIGURE 1:**
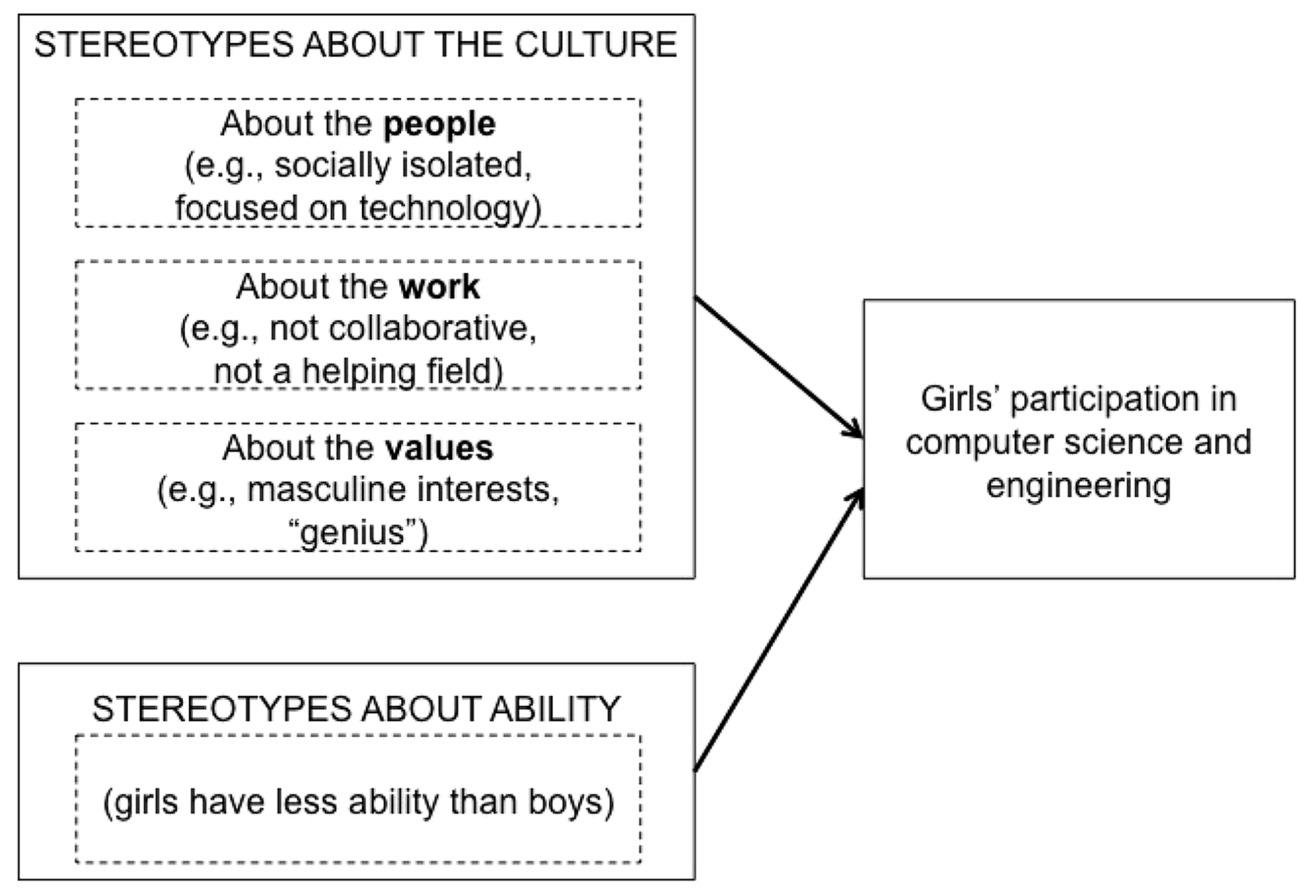
**Students have stereotypes about the culture of computer science and engineering and girls face negative stereotypes about their abilities.** Both types of stereotypes signal to girls that computer science and engineering are not appropriate fields for them.

By elementary school, indeed as early as second grade, girls already hold stereotypes associating boys with math (Cvencek et al., 2011). A large body of research on stereotype threat has investigated the consequences of concerns about being judged through the lens of a negative stereotype (Steele, 1997). This research has shown that negative stereotypes about girls’ math abilities hinder their math performance (Huguet and Regner, 2007; see also Spencer et al., 1999; Master et al., 2014). There are three ways in which the work presented here differs from this established work on stereotype threat. First, work on stereotype threat focuses on stereotypes about girls and women whereas our focus is on students’ stereotypes about the culture of the fields. Both sets of stereotypes – stereotypes about girls themselves and girls’ stereotypes about the culture – may be operating simultaneously to make girls feel like they do not belong in computer science and engineering (see **Figure 1**).

Second, whereas stereotypes about girls’ math abilities (“girls are not good at math”) are negative, we investigate stereotypes that are not always negative (Cheryan et al., 2009). Indeed, stereotypes of computer scientists and engineers can be a source of pride, identification, and belonging for some in the field (e.g., the Geek Girl Dinners organization). This lack of objective negativity can make diversifying how the fields are portrayed more challenging because these stereotypes might not be seen as problematic, even in the face of evidence that many students find them incompatible with how they see themselves. Third, stereotype threat effects are most prominent among women who are already highly identified and invested with STEM, such as STEM majors (Schmader et al., 2008). In contrast, we suggest that stereotypes about the culture preclude many girls from even considering the fields in the first place, and thus deter a larger number of girls from STEM.

## THE ROLE OF STEREOTYPES EARLY IN THE PIPELINE

**FIGURE 2:**
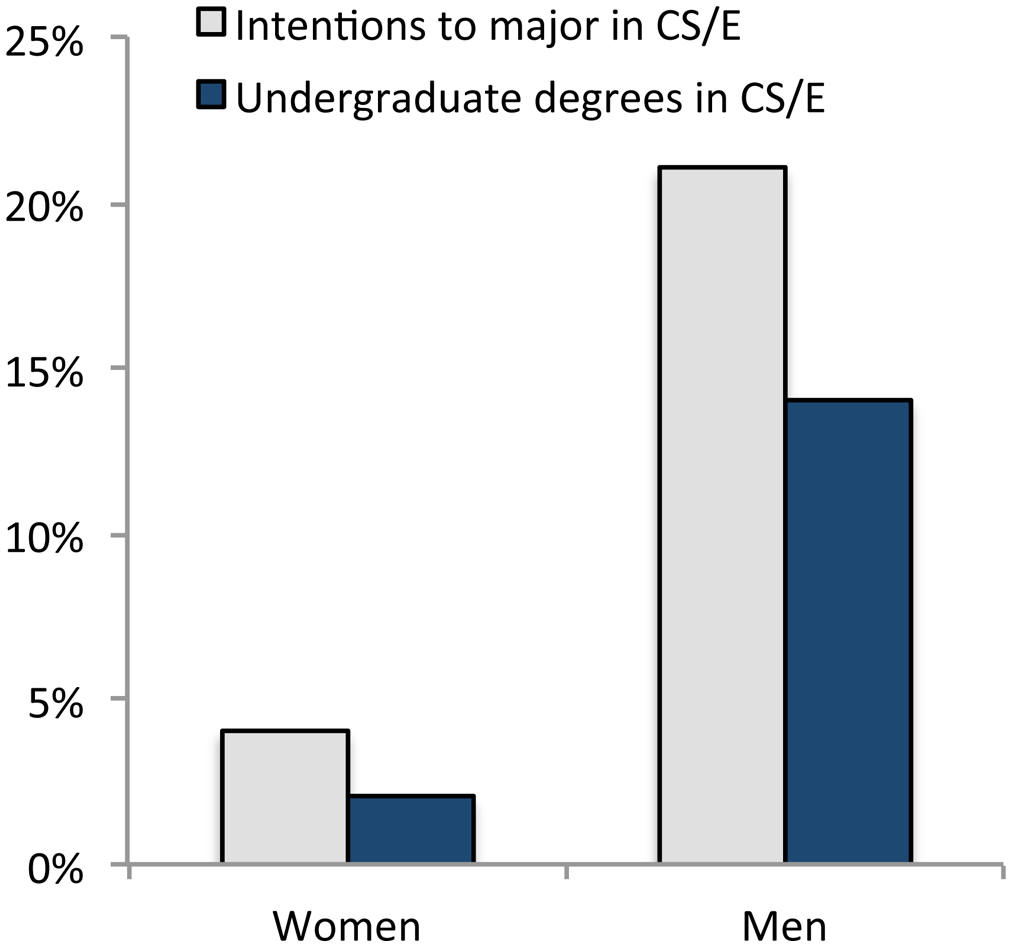
**Percentage of freshmen women and men who intend to major in computer science and engineering, and percentage of undergraduates who graduate with computer science and engineering degrees.** Freshmen data are drawn from U. S. postsecondary institutions while degree data are drawn from U. S. degree-granting institutions eligible to participate in Title IV financial aid programs. The latest available data were used (2010 for freshmen intentions and 2012 for degrees granted). Source: National Science Foundation.

At what juncture in the pipeline are girls and women opting out of computer science and engineering? Although many highly qualified women *leave* these fields (Hewlett et al., 2008), a much larger contributor to the gender gap is that girls are much less likely than boys to *choose them in the first place* (de Cohen and Deterding, 2009). Among high-school students, girls are significantly less likely to take a computer programming class than boys (Shashaani, 1994; Schumacher and Morahan-Martin, 2001), less likely to take the computer science Advanced Placement (AP) test than boys (College Board, 2013), and express less interest in pursuing careers in computer science and engineering than boys (Weisgram and Bigler, 2006). By the time they enter college, men are already more than four times more likely to have an intention to major in computer science and engineering than women (National Science Foundation, 2012). Even if every woman who intended to major in computer science and engineering upon entering college was retained in these fields, men would still be significantly more likely to earn a computer science and engineering degree than women (see **Figure 2**).

Though there is debate on whether biological factors play a role in women’s underrepresentation in STEM (Benbow and Stanley, 1982; Spelke, 2005), differences in interest in computer science and engineering between boys and girls are evident even among students with the highest math abilities. Among the top scorers on a standardized math test administered in the 10th grade, girls relative to boys were more likely to choose social science and health-related majors in college over majors in computer science, engineering, physical sciences, and mathematics (Perez-Felkner et al., 2012). Computer science and engineering are missing out on an entire population of talented girls who are not entering these fields to begin with.

Intervening early in the pipeline (i.e., before college) is important to remedying disparities in computer science and engineering. Societal change will occur only to the extent that the students who are initially drawn into the field are able to remain in it, thus research on retention is, of course, important and useful. However, closing the gender gap in computer science and engineering participation will initially require convincing more girls to join these fields. As we will argue, stereotypes of the culture affect girls’ choices and interest, and do so early in the pipeline.

## WHAT IS THE CONTENT OF COMPUTER SCIENCE AND ENGINEERING STEREOTYPES?

When students think of computer scientists, they often think of “geeky” guys who are socially awkward and infatuated with technology (Mercier et al., 2006; Rommes et al., 2007). The work in computer science and engineering is seen as isolating and relatively dissociated from communal goals such as helping society and working with others (Hoh, 2009; Diekman et al., 2010). Computer scientists and engineers are also perceived as having masculine interests (e.g., playing video games; Cheryan et al., 2011b), and their faculty are more likely than faculty in other fields (e.g., biology, psychology) to believe that an inborn brilliance or genius is required to be successful (Leslie et al., 2015). Of course, many computer scientists do not fit these stereotypes (Borg, 1999). But people’s beliefs have a tremendous power to determine their attitudes, behaviors, and choices, even if these perceptions are completely disconnected from reality (Hasdorf and Cantril, 1954; Ross and Nisbett, 1991). In the words of one female computer science major at Carnegie Mellon, “Oh my gosh, this isn’t for me.’… I don’t dream in code like they do’’ (Margolis et al., 2000, p. 17).

Computer science and engineering stereotypes are pervasive in modern American society and even young students frequently endorse them. When high-school students described computer scientists, the majority (84%) mentioned at least one measurable stereotype, including being technically oriented, singularly focused on technology, socially awkward, masculine, intelligent, or having particular physical traits such as glasses or pale skin (Master et al., unpublished). College students reported similar stereotypes, with 67% mentioning at least one of these stereotypes about computer scientists (Cheryan et al., 2013b). College students were also less likely to believe that computer science and engineering were fields that could be used to help people or work with others than fields such as medicine and law (Hoh, 2009; Diekman et al., 2010).

In today’s society, computer science and engineering stereotypes are perceived as incompatible with qualities that are valued in women, such as being feminine, people-oriented, and modest about one’s abilities (Diekman et al., 2011; Cheryan, 2012; Leslie et al., 2015). As a result, when these stereotypes are prominent, girls and women, but not boys and men, believe that they are dissimilar from those in the field and report a lower “sense of belonging,” or feeling of fit with the culture of the field (Cheryan et al., 2009; Master et al., under review). The less that students feel a sense of belonging in a field, the less likely they are to pursue that field (Good et al., 2012; Smith et al., 2013; Master et al., under review). Changing these stereotypes may allow more girls and women to believe they are welcome in computer science and engineering.

## TRANSMISSION CHANNELS FOR STEREOTYPES ABOUT COMPUTER SCIENCE AND ENGINEERING

Below we review three ways in which students may be exposed to computer science and engineering stereotypes – through media, people in the fields, and environments. Because computer science and engineering are not mandatory and often not even offered in U.S. high schools (Stephenson et al., 2005), many students do not have direct experience with these fields. As a result, students often rely on cultural stereotypes about computer scientists and engineers for knowledge about these fields. However, these stereotype transmission channels have an upside as well: they are particularly well-suited mechanisms of cultural change if interventions are designed appropriately.

## STEREOTYPES TRANSMITTED THROUGH THE MEDIA

Popular movies and television shows like *Real Genius*, *The Big Bang Theory*, and *Silicon Valley* depict computer scientists and engineers as mostly White (and more recently Asian) males, socially unskilled, and singularly obsessed with technology. Similarly, portrayals of technology companies in popular newspapers and books often depict the “startup culture” that infuses some technology and engineering jobs (e.g., Guo, 2014; Miller, 2014). This is unfortunate because in reality such portrayals depict at best only a small percentage of the jobs in computer science and engineering (Bureau of Labor Statistics, 2014). Yet high-school students report that their ideas about what scientists are like are influenced more by the media than by any other source (Steinke et al., 2007). Even brief exposures to television portrayals can influence attitudes toward the group portrayed (Weisbuch et al., 2009).

To examine the extent to which exposure to stereotypical and non-stereotypical media representations influence women’s interest in computer science, women undergraduates read one of two fabricated newspaper articles. One article stated that computer scientists fit the current stereotypes, while the other stated that computer scientists were diversifying and no longer fit the stereotypes. Women who read the stereotypical article expressed less interest in majoring in computer science than women who read the non-stereotypical article. Furthermore, women who read the non-stereotypical article were significantly more interested in computer science than women who read no article (Cheryan et al., 2013b). Changing the images of computer science and engineering in the media may increase women’s interest in these fields.

## STEREOTYPES TRANSMITTED BY NARROW CHARACTERIZATIONS OF PEOPLE IN THE FIELDS

Faculty, students, and industry professionals embody certain characteristics, habits, and belief systems that can signal what is normative and valued in the field. For instance, the National Academy of Engineering’s engineeryourlife.org website features a female computer engineer who appears to fit the definition of a role model for girls: she is successful, competent, and shares their gender (Marx et al., 2005; Stout et al., 2011). However, her profile also describes how she embodies stereotypes of computer scientists and engineers: she started programming at age 11 and works as a Star Wars video game designer. Computer scientists and engineers who embody these stereotypes may discourage women from entering these fields.

To investigate whether encountering a stereotypical computer science student can deter women, undergraduate women were brought into a room to have a conversation with a participant who was actually an actor. Three male and three female actors were used. The conversation was brief – less than 2 min on average – and consisted of the participant and the actor exchanging basic information about themselves (e.g., year, major, hobbies, favorite movie). The actor always stated that he or she was a junior and a computer science major, but half of the participants were randomly assigned to interact with an actor who fit current stereotypes in appearance and preferences (e.g., glasses, t-shirt that said “I code therefore I am,” hobbies that included playing videogames) or one who did not fit these stereotypes (e.g., solid colored t-shirt, hobbies that included hanging out with friends). After the interaction was complete, participants were asked about their interest in their partner’s major and then asked the same questions again 2 weeks later.

Results revealed that women who interacted with the stereotypical student were significantly less interested in majoring in computer science than those who interacted with the non-stereotypical student, and this effect was equally strong regardless of whether the actor was male or female. Moreover, negative effects of stereotypes endured for 2 weeks after the interaction (Cheryan et al., 2013a). The computer science major’s gender mattered less in influencing women’s interest in computer science than the extent to which he or she fit current computer science stereotypes.

Follow-up experiments (a) revealed similar effects of peer stereotypicality on anticipated success in computer science (Cheryan et al., 2011b) and also (b) investigated why people in the field who embody computer science stereotypes may be steering women away from the field. Interacting with a stereotypical computer science major reduced women’s anticipated success in computer science but did not affect men’s anticipated success (Cheryan et al., 2011b). Why? Women felt less similar to the stereotypical student than to the non-stereotypical student, suggesting students may look to other characteristics besides gender when determining with whom they feel similar (see also Cheryan et al., 2011b; Meltzoff, 2013). When the people in computer science depict themselves in a manner consistent with the stereotypes, it can convey to other students that one must fit the stereotypes to be successful in these fields.

Computer scientists and engineers who depict the work in their fields as highly independent may also discourage women from entering their fields. College women who read about an entry-level scientist who spent a typical day doing independent tasks reported less positive attitudes about science careers than college women who read about an entry-level scientist who spent a typical day doing collaborative tasks (Diekman et al., 2011). Moreover, fewer female students are present in fields whose faculty believe that success in their field requires innate brilliance, a belief that is prominent in computer science and engineering (Leslie et al., 2015). Changing stereotypes about the work being isolating and requiring an innate brilliance may draw more women into computer science and engineering.

## STEREOTYPES TRANSMITTED THROUGH ENVIRONMENTS

Objects and environments are powerful because they are seen as providing clues about the dominant culture within that environment, including information about the values, beliefs, norms, and practices (Whiting, 1980; Cialdini et al., 1990; Markus and Kitayama, 1991). Environments that depict computer science and engineering as more compatible with characteristics, interests, and values associated with men and boys are likely to draw fewer girls than boys into them. However, exposing students to computer science and engineering environments that do not fit current male-oriented stereotypes may reduce gender disparities in interest in these fields.

College undergraduates who were not computer science majors (in order to focus on recruitment) entered a classroom in the computer science department at Stanford University, which was decorated in one of two ways (Cheryan et al., 2009). For half the participants, the room had objects that other undergraduates associated highly with computer science majors—Star Trek posters, science fiction books, and stacked soda cans. For the other half of participants, the room contained objects that other undergraduates did not associate with computer science majors—nature posters, neutral books, and water bottles. Women in the room that did not contain the stereotypical objects expressed significantly more interest in majoring in computer science than those in the room that did fit the stereotypes. For men, the environment did not affect their interest in computer science (Cheryan et al., 2009).

Online educational environments are becoming an increasingly important presence in students’ lives as universities use them as tools for education. To test whether the design of virtual classrooms influences educational outcomes, undergraduates virtually entered two classrooms in Second Life, an online 3D interactive virtual environment. Both were introductory computer science classrooms, but one contained stereotypical objects while the other contained non-stereotypical objects. Whereas only 18% of women chose to take the course in the stereotypical classroom, more than half of men (60%) chose that classroom. Furthermore, women expected to perform worse in the class with the stereotypical objects than men, but in the non-stereotypical classroom, women’s expectations rose, so that women and men expected to do equally well (Cheryan et al., 2011a).

Why did the stereotypical environment deter women more than men? Women reported a lower sense of ambient belonging in the stereotypical environment, or sense of fit with the material components and with the people assumed to inhabit the environment. In contrast, men reported an equal, and sometimes greater, sense of ambient belonging in the stereotypical environment than the non-stereotypical environment (Cheryan et al., 2009, 2011a). Women were less likely than men to associate themselves with the stereotypical objects, and the more that women perceived the stereotypical environment as masculine, the less interest they expressed in being in that environment (Cheryan et al., 2009).

Earlier in the pipeline, high-school students also show similar effects on their interest in taking introductory computer science in a classroom that fits or does not fit current computer science stereotypes (Master et al., under review). Girls were more likely to choose a non-stereotypical classroom (68% of girls) over a stereotypical one, while boys showed no preference for a non-stereotypical classroom (48%). Moreover, girls’ baseline interest in a computer science course in which the classroom was not described was no different from their interest in a stereotypical course (and both were lower than the non-stereotypical course), suggesting that a stereotypical classroom was consistent with girls’ default assumptions about introductory computer science courses. However, a non-stereotypical environment provided a new image of computer science and increased their interest over baseline. Like their college counterparts, high-school girls felt a lower sense of fit with current computer science stereotypes than did boys. The less that girls reported a sense of fit with the current stereotypes, the more likely they were to be deterred from a stereotypical (but not a non-stereotypical) computer science environment (Master et al., under review). The observed variability between girls is striking and suggests that current stereotypes should be diversified rather than eliminated, a point we discuss in more detail in the next section.

Thus, women and girls may be choosing fields other than computer science and engineering in part due to the constraining power of current stereotypes that portray the culture of the field in a manner that is incompatible with the way that women see themselves. When the constraint is lifted by presenting a non-stereotypical image, girls’ sense of belonging and interest in the field can increase, without reducing boys’ interest.

## THE IMPORTANCE OF VARIABILITY AND DIVERSIFYING PORTRAYALS OF COMPUTER SCIENCE AND ENGINEERING

In all studies investigating effects of stereotypes, there is a sizable portion of students who may be drawn to these fields *because* of these stereotypes. In the studies on environments, some women (typically 20–25% of women) preferred the stereotypical environment over the non-stereotypical environment. Rather than attempting to overhaul current stereotypes, which may deter some men and women, a more effective strategy may be to diversify the image of these fields so that students interested in these fields do not think that they must fit a specific mold to be a successful computer scientist or engineer.

Diversifying the image of computer scientists and engineers may not only attract more women to the field, but also make some men feel more welcome in these fields. Indeed, in the studies on environments, some men (typically 25–30% of men) preferred the non-stereotypical environment over the stereotypical environment. In addition, many men also highly value opportunities to work with and help others (Diekman et al., 2011). Attracting more non-stereotypical men to the field is a way to further stretch stereotypes and diversify a field (Drury et al., 2011).

A question that our readers may have is whether it is fair to present girls with a non-stereotypical image of the fields of computer science and engineering if they will then enter these fields and be unprepared for the male-oriented culture that they encounter there. We believe it is necessary and useful to prepare girls and women for the obstacles they may encounter in male-dominated fields and how to overcome them. We also believe that the cultures of these fields should be changed to be more welcoming of a diversity of people. However, our viewpoint is that girls are currently exposed to an unrealistic image of these fields that depicts all computer science and engineering cultures as fitting a narrow profile. A broader image that shows many different types of people and working environments in computer science and engineering actually represents a more realistic portrayal. Furthermore, once we start the process of welcoming more women and girls into these fields, the process of culture change will likely build on itself and contribute to further improving the actual and perceived culture of these fields for women.

The computer science departments at Carnegie Mellon and Harvey Mudd provide two real-world examples of the power of changing cultural stereotypes to reduce gender disparities in participation. Both increased the proportion of women majoring in computer science from ∼10 to 40% in 5 years (Margolis and Fisher, 2002; Hafner, 2012). In addition to structural changes (e.g., changes in recruiting procedures), both programs changed stereotypes of computer science by using diverse role models, exposing students to a wide range of applications of computer science, and revamping their introductory course so that it was no longer seen as a field only for “geeky know-it-alls” (Margolis and Fisher, 2002; Hafner, 2012). These examples show that efforts to reduce gender disparities in computer science and engineering benefit from actively working to change the culture of these fields, so that they are seen as places where *all* students are valued and have the potential to be successful.

## CONCLUSION: CONTRIBUTIONS TO THEORY AND PRACTICE

Why are girls, even those who grew up with technology in their homes and took advanced math classes in high school, less likely than boys to pursue computer science and engineering? Our central thesis is that girls’ underrepresentation in these fields is not due to their intractable lack of interest in choosing these fields. Instead, we argue that women’s choices are constrained by societal factors, particularly their stereotypes about of the kind of *people,* the *work involved,* and the *values* of these fields (see **Figure 1**). These perceptions, even if they are not accurate, shape the academic choices that girls make by communicating to them where they belong.

We also argue that we can change students’ stereotypes of the culture using relatively simple interventions to environments, the media, and by diversifying the type of people representing these fields. Rather than “de-geeking” the fields, a more successful approach involves creating inclusive cultures so that those who are considering these fields do not necessarily have to embody the stereotypes to believe that they fit there. One concrete way to create inclusive cultures is to consider who is selected to represent the field (e.g., who teaches the introductory courses) and what messages he or she signals about the kind of student who belongs in the field. If all representatives are similar to one another, it can signal that one has to fit that mold in order to be successful in that environment. If there is diversity in who is presented, it sends the message that a variety of people can be successful. Physical spaces are another effective way to signal who belongs. We have shown that it is possible and feasible to create physical spaces within the larger environment that allow both men and women to feel welcome there. Finally, it is also important to change the stories told in the media about these fields and who is found in them.

The main message of this research is that variability is key. Instead of portraying computer science and engineering as narrow fields that are easily stereotyped—and which therefore steer a large number of students away because they “do not belong”—we can alter how the culture of these fields is represented in the minds of youth. By broadening the mental picture of what it means to be a computer scientist or engineer, we may not only attract more women to these fields, but also be more accurate about what computer science and engineering are like and what they have the potential to become.

## Conflict of Interest Statement

The authors declare that the research was conducted in the absence of any commercial or financial relationships that could be construed as a potential conflict of interest.
